# RNA-Seq Analysis of the Key Long Noncoding RNAs and mRNAs Related to the Regulation of Acute Heat Stress in Rainbow Trout

**DOI:** 10.3390/ani12030325

**Published:** 2022-01-29

**Authors:** Chang-Qing Zhou, Wei Ka, Hui-Jun Zhang, Ya-Lan Li, Pan Gao, Rui-Jun Long, Shun-Wen Yang, Jian-Lin Wang

**Affiliations:** 1 State Key Laboratory of Grassland Agro-Ecosystems, Key Laboratory of Grassland Livestock Industry Innovation, Ministry of Agriculture and Rural Affairs, Grassland Agriculture Engineering Center, Ministry of Education, College of Pastoral Agriculture Science and Technology, Lanzhou University, Lanzhou 730020, China; zhoucq@lzu.edu.cn (C.-Q.Z.); gaop20@lzu.edu.cn (P.G.); 2 School of Life Sciences, Lanzhou University, Lanzhou 730000, China; longrj@lzu.edu.cn; 3 Gansu Fishery Research Institute, Lanzhou 730000, China; kw15346802471@163.com; 4 Gansu Agriculture Technology College, Lanzhou 730000, China; huijun2007an@163.com (H.-J.Z.); liyalan0819@163.com (Y.-L.L.)

**Keywords:** rainbow trout, heat stress, head kidney, transcriptome

## Abstract

**Simple Summary:**

At present, climate warming is a very serious environmental problem. A sudden and large increase or decrease in temperature is likely to cause stress response in animals. Rainbow trout is a kind of cultured cold-water fish, which is very sensitive to high temperature. Therefore, it is very vulnerable to heat waves during production. The current study found that the behavior, antioxidant capacity, and natural immune function of rainbow trout under acute heat stress were significantly enhanced in the early stages of stress response, but its anti-stress ability decreased with an increase in stress intensity and duration. Transcriptome sequencing and bioinformatics analysis showed that some non-coding RNAs could competitively bind to target genes, and jointly participate in metabolism, apoptosis, and the immune regulation of rainbow trout under stress environments. In conclusion, our study can lay a theoretical foundation for the breeding of heat-resistant rainbow trout varieties.

**Abstract:**

As the global climate warms, more creatures are threatened by high temperatures, especially cold-water fish such as rainbow trout. Evidence has demonstrated that long noncoding RNAs (lncRNAs) play a pivotal role in regulating heat stress in animals, but we have little understanding of this regulatory mechanism. The present study aimed to identify potential key lncRNAs involved in regulating acute heat stress in rainbow trout. lncRNA and mRNA expression profiles of rainbow trout head kidney were analyzed via high-throughput RNA sequencing, which exhibited that 1256 lncRNAs (802 up-regulation, 454 down-regulation) and 604 mRNAs (353 up-regulation, 251 down-regulation) were differentially expressed. These differentially expressed genes were confirmed to be primarily associated with immune regulation, apoptosis, and metabolic process signaling pathways through Gene Ontology and Kyoto Encyclopedia of Genes and Genomes pathway enrichment analysis and coding-noncoding co-expression network analysis. These results suggested that 18 key lncRNA-mRNA pairs are essential in regulating acute heat stress in rainbow trout. Overall, these analyses showed the effects of heat stress on various physiological functions in rainbow trout at the transcriptome level, providing a theoretical basis for improving the production and breeding of rainbow trout and the selection of new heat-resistant varieties.

## 1. Introduction

Rainbow trout are cold-water fish that are widely farmed worldwide. They have no obvious lower limit of temperature, 0–18 °C is their optimum growth temperature, and they are extremely sensitive to high temperature. When the water temperature exceeds 20 °C, it will cause a series of obvious stress reactions, such as reduced or decreased intake, abnormal behaviors, growth and reproduction disorders, and immunosuppression [1]. In water, their body temperature is close to the temperature of the surrounding water. When they cannot stand the high temperature of the surrounding environment, some fish can migrate to more profound and colder water, which can help the animals to a certain extent. However, for most fish, high temperatures mean misfortune. In extreme weather, shallow water will heat up rapidly (especially in intensive artificial environments), making the ambient temperature of rainbow trout close to the extreme temperature they can withstand. This makes the rainbow trout an ideal test species for studying whether physiological regulation can keep up with rising temperatures.

The head kidney is an organ similar to the mammalian adrenal gland that synthesizes and releases hormones and produces lymphocytes [2,3]. In terms of function, the head kidney, on the one hand, produces lymphocytes and can synthesize and release cytokines, which has the function of the immune system; on the other hand, endocrine organs that produce and release hormones participate in the regulation of stress and physiological activities by secreting cortisol, catecholamine, and thyroid hormone. The unique function and action form of head kidney makes the bi-directional information exchange and transmission between immunity and endocrine become more close and complicated [2]. Continuous and intense stress stimulation will cause the animal’s body to produce many free radicals and reactive oxygen species (ROS), which damage lipid substances in the cell membrane. Some lipid decomposition products will seriously affect the metabolism and functional activities of normal cells, resulting in cell dysfunction, and then cell damage. The detection of antioxidant factors in rainbow trout serum, including superoxide dismutase (SOD), malondialdehyde (MDA), total antioxidant capacity (T-AOC), and Na^+^-K^+^-ATPase, can directly reflect the state of the immune and antioxidant systems of the animal and can also indirectly reflect the degree of peroxidation damage in the body. Under heat stress, macrophages in the head kidney are activated and secrete a large number of pro-inflammatory cytokines, including lysozyme (LYZ), tumor necrosis factor alpha (TNF-α), interleukin 1β (IL-1β), and interleukin 6 (IL-6). In stress response, the release of pro-inflammatory cytokines is an important cause of stress injury and inflammatory response [2,4].

Transcriptomics are the collection of genes studied at the RNA level in a particular tissue or a population of cells at a specific developmental stage or functional state. The transcriptome is essentially different from the genome. Under the influence of internal and external environment, transcriptomes can reflect the functional state and phenotype of animal cells in real time and accurately. Therefore, transcriptomics can be used to explore the real-time dynamic linkages between animal genomes and environmental interactions [5]. Noncoding RNA (ncRNA) is usually not directly involved in protein translation and is merely a functional RNA molecule transcribed from DNA [6,7]. ncRNAs are subdivided into several subclasses, including microRNAs (miRNAs) and long noncoding RNA (lncRNAs) [8]. These ncRNAs can competitively bind to mRNA and directly or indirectly regulate the biological function of mRNA [9]. lncRNA sequencing uses specific methods to study the sequencing of noncoding RNAs greater than 200 nt in samples, so as to quickly, comprehensively, and accurately obtain the regulation information of the relevant lncRNAs on target gene mRNAs in the animal body under a specific state. lncRNAs usually act as a signal molecule, inducer molecule, lead molecule, and scaffold molecule when involved in the regulation of gene expression. Transcriptome sequencing was used to mine and analyze the genetic information of rainbow trout, and to screen key non-coding genes (lncRNAs, miRNAs) and coding genes (mRNAs) involved in the regulation of acute heat stress for combined analysis. The present study can explore the molecular regulation mechanism of rainbow trout in response to high temperatures, and provide a theoretical basis for the breeding of heat-resistant rainbow trout varieties in the future.

## 2. Materials and Methods

### 2.1. Animals

Norwegian rainbow trout (425-day-old, average weight of 118 ± 5 g) with the same genetic background were selected for the experimental material. The experiment was performed in an indoor recirculating aquaculture system under natural light, and the ammonia nitrogen content in the water was no more than 0.03 mg∙L^−1^, the dissolved oxygen content was no less than 7 mg∙L^−1^, and the pH value was controlled within 7.4 ± 0.2. We used three oxygento pumps (power is 45 watts) to continuously pump air into each tank, increasing the dissolved oxygen in the water. The experiment was conducted after 2 weeks of domestication at 16 °C water temperature, and fasting was performed 24 h before the experiment. The control group (CO) was set based on the optimal growth temperature of rainbow trout being 16 °C. According to our previous research results [1], the 48-h median lethal temperature of rainbow trout is 22.5 °C, set as the treatment group (LTS). We used three sets of electric heating rods and an automatic temperature controller to raise the water temperature to the target temperature within 2 h. After reaching the target temperature (22.5 °C), the acute heat stress model of rainbow trout was established by continuous stress for 24 h. Three biological replications (20 fish per tank) were conducted in both the LTS and CO groups. That is, 60 fish were used in the control group in three different tanks with 20 fish in each tank (n = 3); another 60 fish were also assigned to the acute stress group, again in three tanks, each containing 20 fish (n = 3). At the end of the experiment, rainbow trout were anesthetized using MS-222 (Silver Chemical Laboratories, Redmond, WA, USA). Blood was collected through the caudal vein and serum was prepared. ELISA kit was used to detect various physiological indices in rainbow trout serum. All test kits were provided by Meimian Industrial Co., Ltd. (Yancheng, China). The head kidney tissue was collected for subsequent tests.

### 2.2. Ethics Statement

This study was conducted in strict compliance with animal ethics and ethics, and was reviewed and approved by the Ethics Committee of School of Life Sciences, Lanzhou University (Approval no. EAF2021042).

### 2.3. Transcriptome Sequencing and Analysis

To obtain high-quality sequencing data, we conducted a strict quality control at each step. Total RNA extraction from rainbow trout head kidney was conducted using the mirVana™ miRNA Isolation Kit (Ambion, Austin, TX, USA). The obtained RNA (28S/18S ≥ 1.5 and RNA integrity number > 8.0) was evaluated using an Agilent 2100 (Agilent Technologies, Santa Clara, CA, USA) bioanalyzer. The sequencing of lncRNAs was completed by the Illumina HiSeq platform (Wuhan, China). The construction of cDNA libraries and high-throughput sequencing work refers to our previously published research [10]. Clean reads were obtained by filtering raw data, including the removal of rRNA, low-quality reads, contaminated joints, and reads with high N content in unknown bases were then compared to the reference genome of rainbow trout for transcription assembly. CPC (http://cpc.cbi.pku.edu.cn, Accessed on 29 August 2018), txCdsPredict (http://hgdownload.soe.ucsc.edu/admin/jksrc.zip, Accessed on 29 August 2018), CNCI (https://github.com/www-bioinfo-org/CNCI, Accessed on 29 August 2018) software, and pfam (http://pfam.xfam.org/, Accessed on 29 August 2018) database were used to predict the coding ability of the new transcripts to distinguish between mRNAs and lncRNAs accurately. For quantitative analysis, Bowtie2 (http://bowtie-bio.sourceforge.net/bowtie2/index.shtml, Accessed on 29 August 2018) was used to compare clean reads to the reference sequence, and then RSEM (http://deweylab.biostat.wisc.edu/rsem, Accessed on 29 August 2018) was used to calculate the expression levels of genes and transcripts. The main tool for screening and analyzing differentially expressed genes (DEGs) in current work is the DEGseq software [11]. The significance analysis conditions of the DEGs were fold change ≥ 2 and adjusted *p*-value ≤ 0.001. Cluster analysis was conducted on the expression trend of DEGs using pheatmap software (https://cran.r-project.org/web/packages/pheatmap/index.html, Accessed on 29 August 2018). The Gene Ontology (GO, http://www.geneontology.org, Accessed on 29 August 2018) and the Kyoto Encyclopedia of Genes and Genomes Pathway (KEGG, http://www.genome.jp/kegg/pathway.html, Accessed on 29 August 2018) databases were used to perform functional enrichment analysis on the DEGs, which were divided into three functional modules: biological process (BP); cell component (CC); and molecular function (MF). KEGG analysis identified the DEGs in the signaling pathways. Finally, the Cytoscape software (https://cytoscape.org/, Accessed on 29 August 2018) was used for the joint analysis of the candidate DEGs (lncRNAs, miRNAs, and mRNAs) to obtain the lncRNAs-miRNAs-mRNAs interaction network involved in the regulation of heat stress in rainbow trout.

## 3. Results

### 3.1. Behavior Evaluation

During the experiment, differences in the indices of fish behavior between the control group and different stress temperature groups were observed (Figure S1). When the water temperature was below 22 °C, no fish died in 48 h. When the water temperature reached 22 °C, the fish began to die after 36 h of stress. As the water temperature increased, so did the cumulative number of dead fish. When the water temperature reached 24 °C, the cumulative death rate of rainbow trout in 48 h was almost 100%. After water temperature exceeded 25 °C, the rainbow trout died within hours. This indicates that 22 °C is the limit temperature for rainbow trout to survive under acute temperature rise. At a suitable water temperature (12–18 °C), rainbow trout always swam in neat arrangements, slowly, and in the same direction. In the early stages of heat stress (when the water temperature exceeded 22 °C, within 6 h before the stress), the fish were in an excited state and moved in the upper layer of the tank, showing fidgety actions, increased alertness, and a faster swimming speed. In the middle stages of heat stress (approximately 24 h), the fish were in a state of inhibition, most of them sank to the tank bottom showing reduced activity, and some of the fish lost balance. In the late period of heat stress (approximately 36 h), the fish began to show abdominal upward, increased activity, sometimes jumping out of the water, and even died.

### 3.2. Oxidative Stress Index and Pro-Inflammatory Cytokine Index

**Figure 1:**
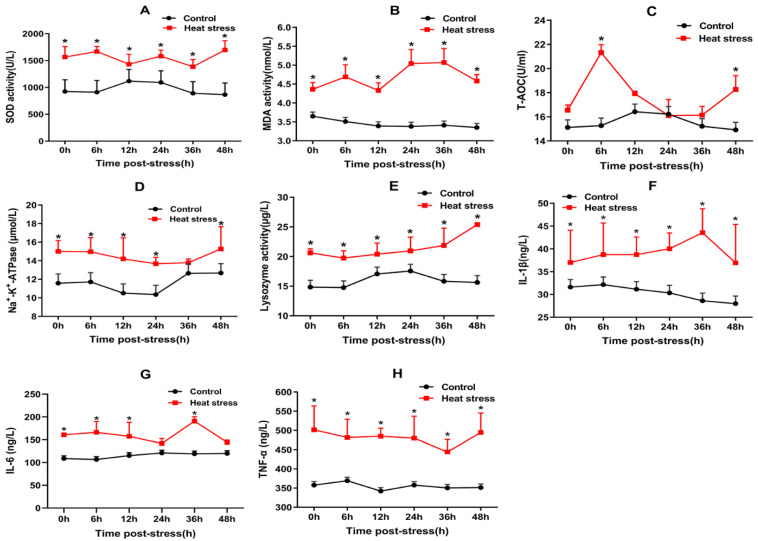
Physiological and biochemical indices of rainbow trout under acute heat stress. (**A**–**D**) represent the levels of antioxidant factors SOD, MDA, T-AOC, and Na^+^-K^+^-ATPase in the serum of rainbow trout under 22.5 °C for 48 h (n = 6, *, *p* < 0.05). (**E**–**H**) represent the levels of inflammatory cytokines lysozyme, IL-1β, IL-6, and TNF-α in the serum of rainbow trout under 22.5 °C for 48 h (n = 6, *, *p* < 0.05).

In the 48-h period post-22.5 °C stress, compared with the CO group, there were significant differences in the SOD activity, MDA content, T-AOC, Na^+^-K^+^-ATPase, LYZ activity, IL-1β, IL-6, and TNF-α in the serum (Figure 1A–H). The SOD activity in the LT group increased significantly from 923.88 to 1565.00 U∙L^−1^ at 0 h and then stayed above 1385.13 U∙L^−1^ for 48 h. The MDA content in the LT group increased significantly from 4.36 nmol∙L^−1^ at 0 h to a maximum of 5.07 nmol∙L^−1^ at 36 h and stayed above 4.33 nmol∙L^−1^ for 48 h. The T-AOC level in the LT group increased significantly from 16.55 U∙mL^−1^ at 0 h to a maximum of 21.32 U∙mL^−1^ after 6 h and stayed above 16.10 U∙mL^−1^ for 48 h. The Na^+^-K^+^-ATPase activity in the LT group increased significantly from 15.00 μmol∙L^−1^ at 0 h to a maximum of 15.26 μmol∙L^−1^ at 48 h and stayed above 13.67 μmol∙L^−1^ for 48 h. The LYZ activity in the LT group increased significantly from 20.62 μg∙L^−1^ at 0 h to a maximum of 25.39 μg∙L^−1^ at 48 h and stayed above 19.74 μg∙L^−1^ for 48 h. The IL-1β level in the LT group increased significantly from 37.02 ng∙L^−1^ at 0 h to a maximum of 43.55 ng∙L^−1^ at 36 h and stayed above 36.93 ng∙L^−1^ for 48 h. The IL-6 level in the LT group increased significantly from 160.73 ng∙L^−1^ at 0 h to a maximum of 190.83 ng∙L^−1^ at 36 h and stayed above 141.86 ng∙L^−1^ for 48 h. The TNF-α level in the LT group increased significantly and reached the peak value of 501.26 ng∙L^−1^ at 0 h and stayed above 444.10 ng∙L^−1^ for 48 h. These values were higher in the LT group than in the CO group at every time point. The significant increase in these biochemical indices indicated oxidative damage in rainbow trout under acute heat stress.

### 3.3. DEGs

**Figure 2:**
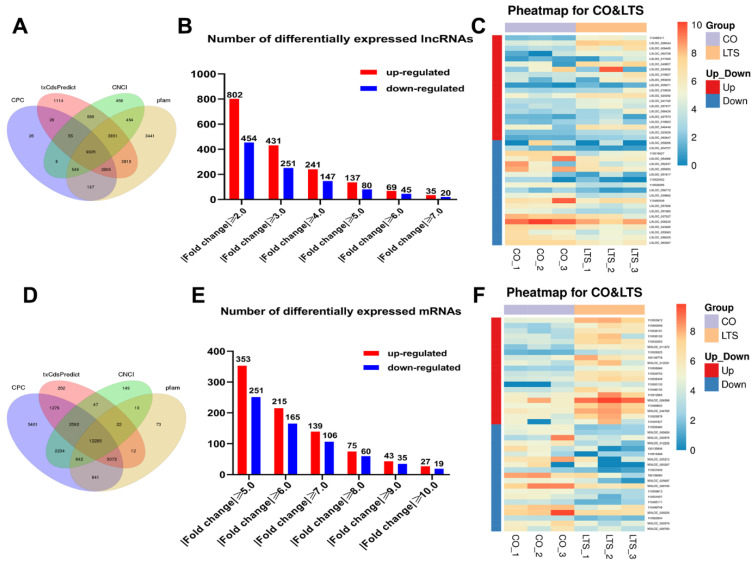
The expression profiling changes of lncRNAs and mRNAs in the head kidney of rainbow trout after post-22.5 °C stress. (**A**) Coding ability predicts outcomes of lncRNAs. (**B**) The statistical results of the number of lncRNAs. (**C**) The heat maps of the top 20 lncRNAs differential genes up-regulated and down-regulated, respectively. (**D**) Coding ability predicts outcomes of mRNAs. (**E**) The statistical results of the number of mRNAs. (**F**) The heat maps of the top 20 mRNAs differential genes up-regulated and down-regulated, respectively.

As stated in our previous study [1], 22.5 °C is the 48-h median lethal temperature (48 h-LT_50_) of rainbow trout under acute heat stress. After 24-h post-22.5 °C heat stress, the head kidney tissue of the rainbow trout was examined to study the molecular regulation mechanism of the heat stress. Six libraries were constructed in this experiment, namely, the control groups (CO-1, CO-2, and CO-3) and the treatment groups (LTS-1, LTS-2, and LTS-3). We used three software predictions (CPC, txCdsPredict, and CNCI) and a protein database (pfam) to predict the coding ability of new transcripts, and identified lncRNAs and mRNAs (Figure 2A,D). According to different differential multiples, the number of selected differential genes was shown in a histogram (Figure 2B,E). Cluster analysis was conducted on the top 20 DEGs up-regulated and down-regulated in lncRNAs and mRNAs (Figure 2C,F). The number of DEGs is shown as follows: there were 55 DE lncRNAs (up-regulated 35, down-regulated 20) obtained when the fold change was ≥7 or ≤-7; there were 46 DE mRNAs (up-regulated 27, down-regulated 19) obtained when the fold change was ≥10 or ≤-10. We presented the information on the top 20 genes in which the DE lncRNAs and mRNAs were up-regulated and down-regulated (Tables S1 and S2). These DEGs may play an essential role in regulating heat stress in rainbow trout, so we focused on their analysis and verification.

### 3.4. Analysis of DEGs

**Figure 3:**
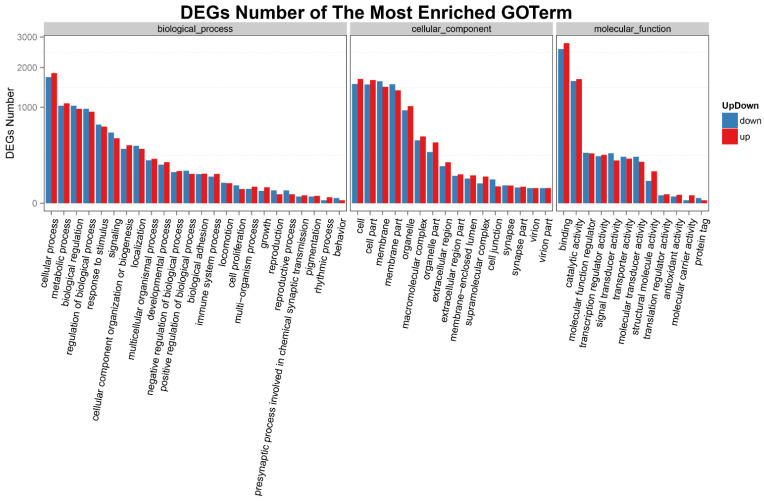
GO analysis of DEGs in the head kidney of rainbow trout after post-22.5 °C stress. The *X*-axis represents the GO functional classification, and the *Y*-axis represents the number of up-regulated or down-regulated genes corresponding to GO term; the red is the number of up-regulated genes, and blue is the number of down-regulated genes.

According to the result of GO functional enrichment, the function and number of DEGs were statistically displayed in the form of a histogram. As shown in Figure 3, DEGs (lncRNAs) in rainbow trout head kidney under acute heat stress were significantly enriched in BP, CC, and MF functional groups. In the BP group, 1084 DEGs enriched in the “metabolic process” were significantly up-regulated and 1034 DEGs were significantly down-regulated; 638 DEGs enriched in “response to stimulus” were significantly up-regulated, and 673 DEGs were significantly down-regulated; 94 DEGs enriched in the “immune system process” were significantly up-regulated, and 77 DEGs were significantly down-regulated. In CC group, 1472 DEGs enriched in “membrane” were significantly up-regulated, and 1614 were significantly down-regulated; 182 DEGs enriched in “extracellular region” were significantly up-regulated, and 150 DEGs were significantly down-regulated; 31 DEGs enriched in “cell junction” were significantly up-regulated, and 62 DEGs were significantly down-regulated. In the MF group, 2779 DEGs enriched in “binding” were significantly up-regulated, and 2579 are significantly down-regulated; 270 DEGs enriched in “molecular function regulator” were significantly up-regulated, and 278 DEGs were significantly down-regulated; 8 DEGs enriched in “antioxidant activity” were significantly up-regulated, and 5 DEGs were significantly down-regulated.

**Figure 4:**
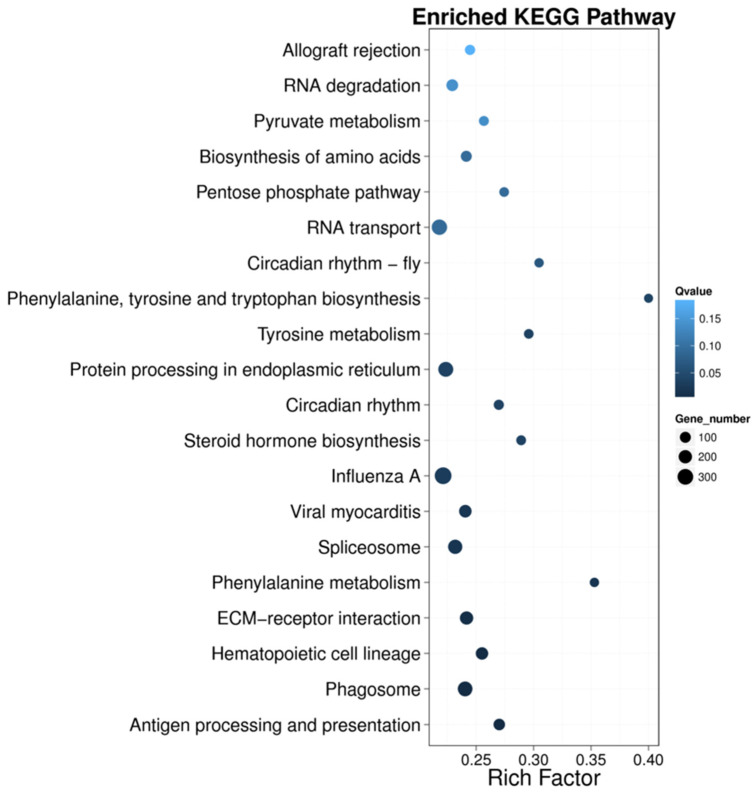
The DEG-enriched KEGG pathway scatter plot in the heat stress regulation of rainbow trout. The *X*-axis represents the enrichment factor, and the *Y*-axis represents the pathway; color represents *p*-value, and dot size represents the number of genes.

Pathway enrichment analysis is a method to classify and analyze multiple genes with a certain biological function according to the form of signaling pathway. In the current study, the top 20 pathways of KEGG enrichment of DEGs in rainbow trout under acute heat stress were presented in the form of scatter plots (Figure 4). The DE lncRNAs in the head kidney of rainbow trout were significantly enriched in the following signaling pathways: allograft rejection, RNA degradation, pyruvate metabolism, biosynthesis of amino acids, the pentose phosphate pathway, RNA transport, circadian rhythm-fly, phenylalanine, tyrosine and tryptophan biosynthesis, tyrosine metabolism, protein processing in the endoplasmic reticulum (ER), circadian rhythm, steroid hormone biosynthesis, influenza A, viral myocarditis, spliceosome, phenylalanine metabolism, ECM-receptor interaction, hematopoietic cell lineage, phagosome, and antigen processing and presentation. These results were similar to enriched pathways, which were analyzed with predicted genes in the heat stress regulation of rainbow trout.

### 3.5. Regulatory Network of Analysis

**Figure 5:**
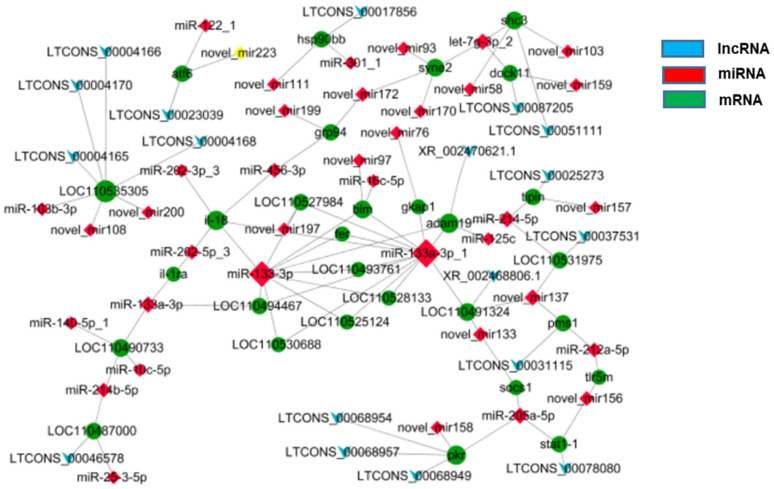
Interacting network analysis of lncRNA-miRNA-mRNA in the head kidney of rainbow trout after post-22.5 °C stress.

In line with previous miRNA sequencing data [1], the lncRNA-miRNA-mRNA regulatory network was constructed to conduct interaction association analysis on the major DEGs involved in regulating heat stress in rainbow trout. The results showed that there were 18 lncRNAs, 38 miRNAs, and 29 mRNAs DEGs co-expressed, among which there were 88 miRNA-mRNA interaction pairs and 18 lncRNA-mRNA interaction pairs (Figure 5).

## 4. Discussion

**Figure 6:**
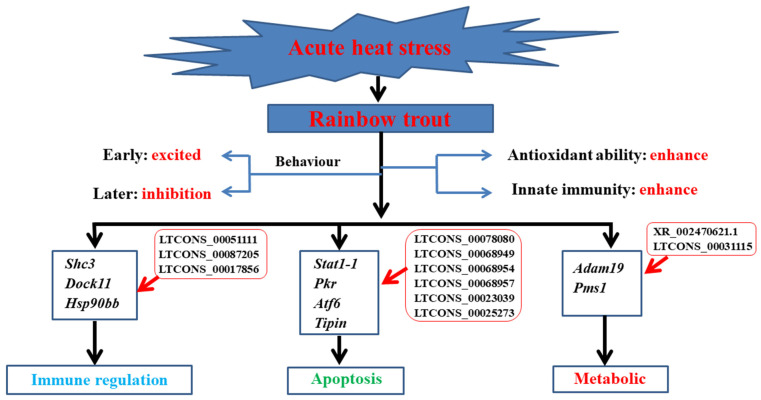
Diagram of regulated pathways in head kidney of rainbow trout exposed to 22.5 °C heat stress for 24 h. The pathways involved in immune regulation, metabolic, and apoptotic.

Global warming and intensification have begun to affect the survival and reproduction of an increasing number of animals, especially cold-water fish such as rainbow trout. The stress of the high temperature environment on animals has a cascade effect from molecules, cells, and organs to the whole body, which destroys the homeostasis of the internal environment of the body and leads to the death of the fish [12]. The damage caused by non-biological stress is procedural. Studies have shown that when stress intensity and duration exceed the threshold of animal tolerance, “metabolic compensation strategy” will be transformed into “metabolic conservation strategy”, resulting in stress damage to some organs, this is to endanger the organs that will not affect life activities temporarily [13]. It is an indisputable fact that high temperatures can damage the internal environment of fish. Although many studies reported that heat stress affects this behavior, energy, metabolism, growth, reproduction, immunity, disease resistance, and other functions of fish, the molecular regulation mechanism of heat stress on fish is still not clear. In the present study, when exposed to 22.5 °C for 48 h, the levels of antioxidant factors (including SOD, MDA, T-AOC, and Na^+^-K^+^-ATPase) and inflammatory cytokines (including LYZ, TNF-α, IL-1β, and IL-6) in serum were significantly increased compared with the control group. These results indicated that although the innate immune function of rainbow trout might be enhanced in the early stages of acute heat stress, lipid metabolism would be impaired with the accumulation of many peroxides, which leads to oxidative damage. Transcriptome sequencing studies showed that acute heat stress had the most pronounced effects on the organism’s immune, metabolic, and apoptotic functions (Figure 6).

### 4.1. Immune Regulation

In the current study, transcriptome study and analysis were performed on the head kidney of rainbow trout under acute heat stress, and comprehensive bioinformatics analysis was performed on differentially expressed lncRNAs and mRNAs. Bioinformatics analysis shows that immune regulation plays an important role in rainbow trout resistance to acute heat stress. Meanwhile, DEGs-related to immune regulation were enriched in antigen processing and presentation, and toll-like receptor signaling pathways. Under acute heat stress, transcriptional sequencing showed that DEGs lncRNAs (LTCONS_00051111) and miRNAs (let-7g-3p_2, novel_mir103, and novel_mir58) were significantly down-regulated in the head kidney of rainbow trout, and were competitively bound with mRNAs and negatively correlated with the regulation target gene *Shc3* that was significantly up-regulated. Shc proteins are involved in adaptive immune responses [14]. Studies have shown that the expression of the Shc protein is up-regulated in LPS-induced inflammatory response, thereby preventing the secretion and release of pro-inflammatory cytokines (including IL-6 and IL-12) [14]. For example, Shc protein can play an important regulatory role in the production of pro-inflammatory cytokines and anti-inflammatory cytokines in dendritic cells [14]. The dedicator of cytokinesis 11 (*DOCK11*), also known as Zizimin 2, is a guanine nucleotide exchange factor that activates the Rho family of GTPase cell division 42 (*CDC42*) leading to cytoskeleton recombination [15]. As *DOCK11* is similar to *CDC42*, it plays an essential role in humoral immune responses [16,17,18,19]. Sakamoto et al. [20] showed that the lack of *DOCK11* in B cells decreased the frequency of antigen-specific germinal center B cells along with an increase in apoptosis during immunization. In the current study, the DEGs lncRNAs (LTCONS_00087205) and miRNAs (let-7g-3p_2, and novel_mir159) were significantly down-regulated in the head kidney of rainbow trout, were competitively bound with mRNAs, and negatively correlated with the regulation of the target gene *DOCK11* that was significantly up-regulated.

As a highly conserved molecular chaperone, heat shock proteins (*HSPs*) play an essential role in cell function protection, repair, enhancement of stress tolerance and the regulation of innate immune function [12,21,22,23]. In the present study, transcriptome analysis showed that *Hsp90* was highly regulated in the acute heat stress of rainbow trout. The DEGs lncRNAs (LTCONS_00017856) and miRNAs (novel_mir111, and miR-301_1) were significantly up-regulated, competitively bound with mRNAs, and positively correlated with the regulation target gene *HSP90bb* that was significantly up-regulated. The result indicated that in the heat stress environment, the rainbow trout body was induced to produce more *HSPs*, which may be closely related to the key stress signals and apoptosis molecules, and jointly participate in the stress response and immune defense process. In related studies, *Hsp90* is often considered as a marker of cellular stress and endogenous protective proteins, and has the function of molecular chaperone [24]. Other studies showed that in the early stage of heat stress (4 h), the expression of *HSPs* in rainbow trout erythrocytes was up-regulated, which could prevent cell apoptosis, and promote cell survival [25]. However, when the intensity and duration of stress increases, *HSPs* can no longer repair the damage and maintain the stability of the intracellular environment, and therefore promote cell death through necrosis or apoptosis. There are many similar results in fish studies. The expression of *Hsp90* mRNA is significantly up-regulated in various organs under heat stress [12,21]. These results indicate that the *Hsp90* gene is not only a marker of heat-stress response, but also plays an important role in anti-stress damage and immune regulation.

### 4.2. Apoptosis

Signal transducer and activator transcription (*STAT*) play essential roles in cell growth, differentiation, proliferation and apoptosis [26]. *STAT* binds to its receptor, activates Janus kinase (*JAK*), and transits cell signals from extracellular to intracellular via the JAK-STAT pathway, activating relevant genes and realizing the regulation of cell growth and differentiation [26]. Ouchi et al. [27] believed that most of the activity of *IFN-γ* was the result of the transcriptional response mediated by *STAT1*; under the synergistic effect of *BRCA1* tumor suppressor and *STAT1*, the transcription of the *IFN-γ* target gene subsets could be differentially activated, and this cytokine could mediate growth inhibition. In the present study, the DEGs lncRNAs (LTCONS_00078080) and miRNAs (miR-205a-5p and novel_mir156) were significantly up-regulated, and were competitively bound with mRNAs and negatively correlated with the regulation target gene *Stat1-1* that was significantly down-regulated.

Protein kinase R (*PKR*) is considered to be an interferon-induced protein that plays an important role in antiviral, antitumor and anticellular activities, as well as in the regulation of immunity and apoptosis [28]. *PKR* can be activated by factors such as *HSPs*, growth factors, and heparin, and can also be activated in response to various insults, including non-viral pathogens, nutrient or excess energy, cytokines, calcium, ROS, radiation and multiple stressors resulting from the presence of a large quantity of unfolded proteins [28,29,30,31,32]. Similarly, *PKR* is not only involved in the activation of several transcription factors, but also participates in stimulus-induced apoptosis, including LPS and cytokines [28]. Therefore, *PKR*, as a central hub of cell stress signal detection and response, plays an important role in signal transmission and regulation of cell functions during the stress response [33]. In our study, the DEGs lncRNAs (LTCONS_00068949, LTCONS_00068954, and LTCONS_00068957) and miRNAs (miR-205a-5p and novel_mir158) were significantly down-regulated, and were competitively bound with mRNAs and negatively correlated with the regulation target gene *PKR* that was significantly up-regulated. Thus, the expression of some apoptosis-inducing genes under heat stress is regulated by *PKR*. These results indicate that *PKR* may be a key gene involved in the regulation of apoptosis under heat stress, which needs to be focused and verified.

Activating transcription factor 6 (*ATF6*) is a member of the leucine zipper family of transcription factors and ER-locator protein [34]. *ATF6* can transduce stress signals to the ER and be a major regulator of organogenesis and tissue homeostasis [34]. When additional protein synthesis is triggered in the ER, *ATF6*, with the ER stress transduction factor inositol required enzyme 1 and *PKR*-like endoplasmic reticulum kinase, can slow protein translation and induce stress to reduce chaperone and folding enzyme production [34]. In the current study, the DEGs lncRNAs (LTCONS_00023039) and miRNAs (miR-122_1 and novel_mir223) were significantly down-regulated, and were competitively bound with mRNAs and negatively correlated with the regulation target gene *ATF6* that was significantly up-regulated.

TIPIN protein is a conserved replication-related protein, which can prevent the collapse of the replication fork and play a role in slowing the replication process [35]. TIPIN and its partner TIM (Timeless) are part of the fork protection complex and participate in normal DNA replication to maintain genomic stability [36]. In the current study, the DEGs lncRNAs (LTCONS_00025273) and miRNAs (miR-214-5p and novel_mir157) were significantly up-regulated, and were competitively bound with mRNAs and negatively correlated with the regulation target gene TIPIN that was significantly down-regulated. Chou et al. [37] showed that TIPIN is a nuclear protein that associates with the replicative helicase and protects cells against genotoxic agents. In the studies related to DNA damage, TIPIN is considered to be important in the cell cycle arrest and maintenance of DNA replication, and the depletion of TIPIN can lead to apoptosis in breast cancer cells [38]. Current studies suggest that apoptosis-related DEGs play an important role in the regulation of acute heat stress, which requires further verification and analysis.

### 4.3. Metabolic

Some studies suggest that the effect of heat stress on the metabolic function of animals has nothing to do with the decrease in food intake, but is closely related to the metabolic changes of carbohydrates, lipids, and proteins after absorption [39]. Zhao et al. [40] found that six key genes, including lysophosphatidylcholine acyltransferase (*Lpcat2*), ethanolamine kinase 1 (*Etnk1*), tafazzin (*Taz*), sterol carrier protein 2 (*Scp2*), cholesterol 25-hydroxylase-like protein (*Ch25hl*), and glycerol-3-phosphate dehydrogenase (*Gpd1l*), were related to lipid metabolism regulation in turbot kidney under heat stress through integrated analysis of metabolome and transcriptome. A disintegrin and metalloproteinases (ADAMs) are proteolytic enzymes that regulate cell phenotypes by affecting cell adhesion, migration, proteolysis, and signal transduction [41]. Recent studies have found that the elevated expression of *ADAM19* is related to the occurrence of metabolic syndrome, and that it is expected to become a new target for treating metabolic syndrome in humans and mice [42]. In the current study, the DEGs lncRNAs (XR_002470621.1) and miRNAs (miR-125c, miR-133-3p, miR-133a-3p_1, and miR-214-5p) were significantly up-regulated, were competitively bound with mRNAs, and negatively correlated with the regulation target gene *ADAM19* that was significantly down-regulated. *PMS1* is a mismatch repair gene that plays an important role in cancer and some genetic diseases [43]. Mismatch repair is a highly conserved DNA mismatch repair protein that occurs frequently during DNA replication, gene recombination, and some damage, leading to the recognition and repair of mismatched bases [44]. As one of the mismatch repair genes, *PMS1* has been widely studied in repairing DNA damage and carcinogenesis, but seldom in stress regulation and metabolism. In the current study, the DEGs lncRNAs (LTCONS_00031115) and miRNAs (miR-212a-5p and novel_mir137) were significantly up-regulated in the head kidney of rainbow trout, and were competitively bound with mRNAs and negatively correlated with the regulation target gene *PMS1*, which was significantly down-regulated. In conclusion, ncRNA and mRNA competitively bind in correlation analysis, and jointly regulate target genes to participate in the regulation of acute heat stress in rainbow trout.

## 5. Conclusions

Conclusively, the present study provides a systematic description of the changes in lncRNAs and mRNAs in rainbow trout under acute heat stress conditions. Within 48 h of acute heat stress, oxidative damage was observed in rainbow trout at the physiological level due to lipid metabolism disorders and the excessive release of pro-inflammatory cytokines. At the transcriptome level, the DEGs lncRNAs and miRNAs competitively bind to mRNAs. The target genes are mainly involved in physiological functions such as immune regulation, apoptosis, and metabolic processes in rainbow trout. Furthermore, the data obtained represent a resource for further investigations of the function of some of these ncRNAs, as it could provide the basic information required to elucidate the mechanisms associated with regulating acute heat stress in rainbow trout at the molecular level.

## Data Availability

The data presented in this study are available on request from the corresponding author.

## Supplementary Materials

The following supporting information can be downloaded at: https://www.mdpi.com/article/10.3390/ani12030325/s1, Figure S1: Behavior changes of rainbow trout under heat stress for 48 h. A. Behavior of rainbow trout at 16 °C water temperature; B. Behavior of rainbow trout at 22.5 °C stress for 6 h; C. Behavior of rainbow trout at 22.5 °C stress for 24 h; D. Behavior of rainbow trout at 22.5 °C stress for 48 h; Table S1: The detail information of the top 20 up-regulated and 20 down-regulated lncRNAs; Table S2: The detail information of the top 20 up-regulated and 20 down-regulated mRNAs.
