# Supplementary material for: RNA-Seq Analysis of the Key Long Noncoding RNAs and mRNAs Related to the Regulation of Acute Heat Stress in Rainbow Trout
Source: Animals (Basel). 2022 Jan 29;12(3):325. doi: 10.3390/ani12030325 (PMC8833469; doi:10.3390/ani12030325)
Supplement: Supplementary file 1 [file animals-12-00325-s001.zip › animals-1528258-supplementary.pdf]

Behavior evaluation

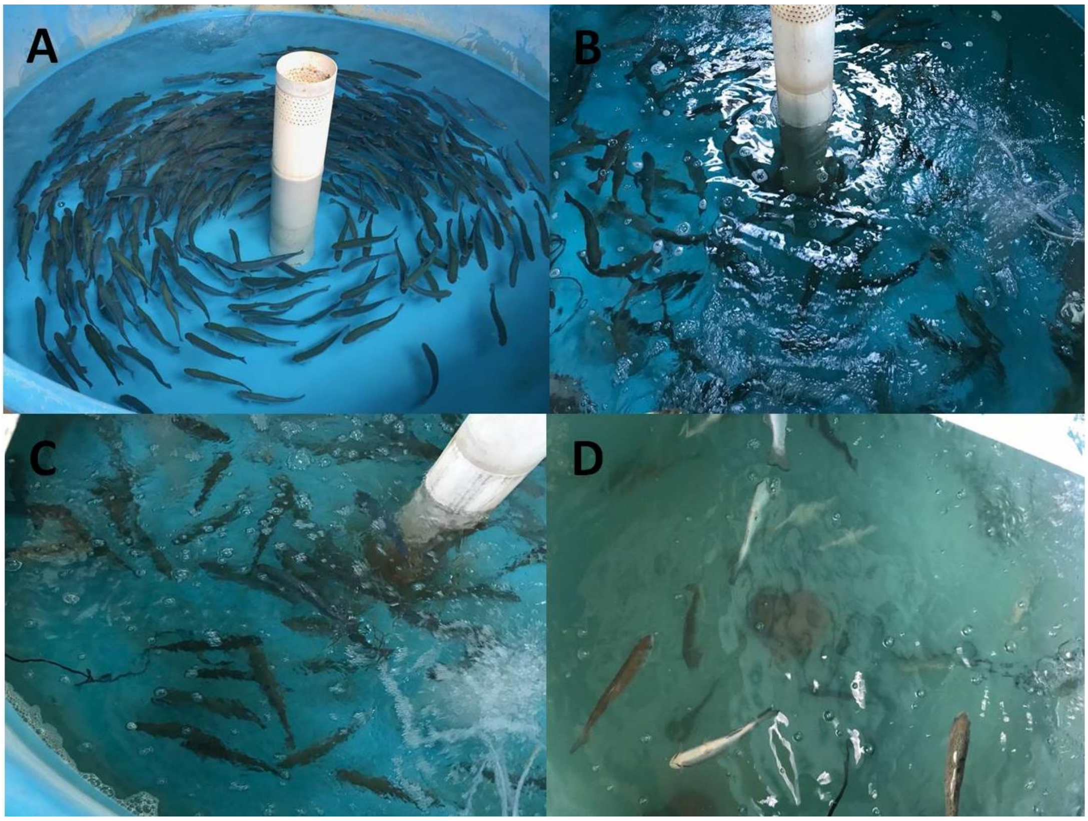

Figure S1. Behavior changes of rainbow trout under heat stress for 48 h. A. Behavior of rainbow trout at 16 ℃ water temperature; B. Behavior of rainbow trout at 22.5 ℃ stress for 6 h; C. Behavior of rainbow trout at 22.5 ℃ stress for 24 h; D. Behavior of rainbow trout at 22.5 ℃ stress for 48 h.

DEGs:

Table S1. The detail information of the top 20 up-regulated and 20 down-regulated lncRNAs.

GeneID	length	CO-FPKM	LTS-FPKM	log2Ratio(LTS/CO)	Regulation	p-value	q-value
LXLOC_060073	495.19	184.29	3018.04	4.049188	Up	0.00	0.00
110485411	2107.79	630.06	9221.18	3.887016	Up	0.00	0.00
LXLOC_052153	3658	225.96	3048.46	3.769567	Up	0.00	0.00
LXLOC_033774	748	115	1164	3.355011	Up	0.00	0.00
LXLOC_052896	1250.14	108	976.77	3.192613	Up	0.00	0.00
LXLOC_026044	887.29	2011.76	17548.68	3.140458	Up	0.00	0.00
LXLOC_006485	1238.9	1086	8651	3.009468	Up	0.00	0.00
LXLOC_050708	1223	660.71	4200.13	2.68397	Up	0.00	0.00
LXLOC_022906	1821	110	695	2.675135	Up	0.00	0.00
LXLOC_017325	4582	724.69	4555.25	2.66772	Up	0.00	0.00

110508587	1696	107.18	637.52	2.58806	Up	0.00	0.00
LXLOC_018940	3808.93	458	2642	2.543837	Up	0.00	0.00
LXLOC_049857	334.87	588	3336	2.519857	Up	0.00	0.00
LXLOC_012717	504	221	1253	2.518894	Up	0.00	0.00
LXLOC_052154	2054.53	294.91	1668.19	2.515563	Up	0.00	0.00
LXLOC_040732	8309	107.47	594.08	2.482349	Up	0.00	0.00
LXLOC_012650	462.76	200	1095	2.468485	Up	0.00	0.00
LXLOC_042478	5122	198	1068	2.446965	Up	0.00	0.00
LXLOC_059453	948	100.52	521.8	2.39164	Up	0.00	0.00
LXLOC_015190	8622	140	709.9	2.357815	Up	0.00	0.00
LXLOC_053890	413.44	2694.85	151.5	-4.13719	Down	0.00	0.00
LXLOC_059206	1259.36	8589.49	617.64	-3.78211	Down	0.00	0.00
LXLOC_051097	231.87	1228.31	109	-3.47865	Down	0.00	0.00
110518965	1304.97	1458.04	130.29	-3.46861	Down	0.00	0.00
LXLOC_051689	1077.34	984.55	105.11	-3.21194	Down	0.00	0.00
LXLOC_001875	565	2913.19	338.51	-3.0897	Down	0.00	0.00
LXLOC_057121	1441	579.4	101.98	-2.49065	Down	0.00	0.00
LXLOC_004757	9737.56	5064.01	1056.11	-2.24589	Down	0.00	0.00
LXLOC_059952	896	496.38	106.57	-2.20402	Down	0.00	0.00
LXLOC_056449	1386	1076.25	233.43	-2.18933	Down	0.00	0.00
110516627	1099.72	15729.97	3415.27	-2.18782	Down	0.00	0.00
LXLOC_054066	666.78	17071.52	3734.96	-2.1768	Down	0.00	0.00
LXLOC_014106	5751	1188	260	-2.17633	Down	0.00	0.00
LXLOC_054127	1539	950.2	216.16	-2.12051	Down	0.00	0.00
LXLOC_031067	5396	988.58	227.13	-2.10621	Down	0.00	0.00
LXLOC_046978	695	575	134	-2.0857	Down	0.00	0.00
LXLOC_053611	493	439	103	-2.07595	Down	0.00	0.00
LXLOC_014755	1104	1022	243	-2.05674	Down	0.00	0.00
110499932	407	2068.92	493.7	-2.05155	Down	0.00	0.00
LXLOC_052656	1180	1449	356.48	-2.00754	Down	0.00	0.00

Table S2. The detail information of the top 20 up-regulated and 20 down-regulated mRNAs.

Gene ID	length	CO-FPKM	LTS-FPKM	log2 Ratio	Regulation	p-value	q-value
110522488	2743	337	68274.99	7.67809	Up	0.00	0.00
110529844	2740	616	79360	7.024963	Up	0.00	0.00
110504077	2179.65	264	28562	6.773041	Up	0.00	0.00
MXLOC_008017	10821	101.34	6802.59	6.084434	Up	0.00	0.00
100135844	2097	704	36876	5.726589	Up	0.00	0.00
110512845	716	375.9	16636.3	5.483467	Up	0.00	0.00
110521132	2193	375	13956	5.233477	Up	0.00	0.00
110502857	2170	192.67	4498.95	4.56101	Up	0.00	0.00
110494097	2974	897	18731	4.399802	Up	0.00	0.00
110522086	9938.91	378.57	6819.41	4.186641	Up	0.00	0.00
110533369	2233.98	139	2268.01	4.043896	Up	0.00	0.00

MXLOC_005184	6334	232.75	3518.84	3.933872	Up	0.00	0.00
MXLOC_022027	4666	104.13	1282.65	3.638295	Up	0.00	0.00
110501731	2233	363.55	4024.05	3.484048	Up	0.00	0.00
MXLOC_038395	3415.17	286.27	2974.06	3.392611	Up	0.00	0.00
100135836	2785	524.38	5258.77	3.341666	Up	0.00	0.00
110510079	1398	319.17	3010.39	3.253179	Up	0.00	0.00
110496159	24131	615.62	5799.68	3.251487	Up	0.00	0.00
110518068	5141	103.68	974.53	3.248195	Up	0.00	0.00
110503972	2478	6992.92	63839.62	3.206111	Up	0.00	0.00
110521964	1563	3625.35	113.66	-4.9797	Down	0.00	0.00
110508773	8867.12	4784.44	220.63	-4.42302	Down	0.00	0.00
MXLOC_001882	1632	2209.15	116.39	-4.23083	Down	0.00	0.00
110524259	1663.55	10098.95	663.28	-3.91282	Down	0.00	0.00
MXLOC_016984	4266.69	6175	424	-3.84868	Down	0.00	0.00
110522444	4782.83	9489.56	739.54	-3.66602	Down	0.00	0.00
MXLOC_031155	1131.44	1617.83	126.53	-3.66088	Down	0.00	0.00
110526114	4320	8424.25	737.64	-3.49793	Down	0.00	0.00
110504783	2716	1391.89	123.36	-3.48047	Down	0.00	0.00
110520578	5422.52	2033.46	197.42	-3.34897	Down	0.00	0.00
MXLOC_017187	3349	3247.49	350.11	-3.19782	Down	0.00	0.00
MXLOC_060831	3306	4710.6	540.25	-3.10859	Down	0.00	0.00
110526460	5748.27	20926.25	2413.44	-3.10053	Down	0.00	0.00
MXLOC_018990	3265	2505.56	298.99	-3.05134	Down	0.00	0.00
MXLOC_025584	396	5711.67	691.62	-3.03024	Down	0.00	0.00
MXLOC_005650	3489.61	11983.76	1453.96	-3.0274	Down	0.00	0.00
MXLOC_013525	2490.66	7635.68	988.74	-2.93347	Down	0.00	0.00
110529302	1494.57	4628.27	605.74	-2.91808	Down	0.00	0.00
100136743	1710	6355	858	-2.87322	Down	0.00	0.00
110533228	2949	877.27	124.13	-2.80554	Down	0.00	0.00
